# Tim4 deficiency reduces CD301b^+^ macrophage and aggravates periodontitis bone loss

**DOI:** 10.1038/s41368-023-00270-z

**Published:** 2024-02-28

**Authors:** Ziming Wang, Hao Zeng, Can Wang, Jiaolong Wang, Jing Zhang, Shuyuan Qu, Yue Han, Liu Yang, Yueqi Ni, Wenan Peng, Huan Liu, Hua Tang, Qin Zhao, Yufeng Zhang

**Affiliations:** 1 State Key Laboratory of Oral & Maxillofacial Reconstruction and Regeneration, Key Laboratory of Oral Biomedicine Ministry of Education, Hubei Key Laboratory of Stomatology, School & Hospital of Stomatology, Taikang Center for Life and Medical Sciences, Wuhan University, Wuhan, China; 2 School of Stomatology, Nanchang University, Nanchang, China; 3 Institute of Infection and Immunity, Science and Technology Innovation Center, Shandong First Medical University & Shandong Academy of Medical Sciences, Jinan, China; 4 Medical Research Institute, School of Medicine, Wuhan University, Wuhan, China

**Keywords:** Periodontitis, Mechanisms of disease

## Abstract

Periodontitis is a common chronic inflammatory disease that causes the periodontal bone destruction and may ultimately result in tooth loss. With the progression of periodontitis, the osteoimmunology microenvironment in periodontitis is damaged and leads to the formation of pathological alveolar bone resorption. CD301b^+^ macrophages are specific to the osteoimmunology microenvironment, and are emerging as vital booster for conducting bone regeneration. However, the key upstream targets of CD301b^+^ macrophages and their potential mechanism in periodontitis remain elusive. In this study, we concentrated on the role of Tim4, a latent upstream regulator of CD301b^+^ macrophages. We first demonstrated that the transcription level of *Timd4* (gene name of Tim4) in CD301b^+^ macrophages was significantly upregulated compared to CD301b^−^ macrophages via high-throughput RNA sequencing. Moreover, several Tim4-related functions such as apoptotic cell clearance, phagocytosis and engulfment were positively regulated by CD301b^+^ macrophages. The single-cell RNA sequencing analysis subsequently discovered that *Cd301b* and *Timd4* were specifically co-expressed in macrophages. The following flow cytometric analysis indicated that Tim4 positive expression rates in total macrophages shared highly synchronized dynamic changes with the proportions of CD301b^+^ macrophages as periodontitis progressed. Furthermore, the deficiency of Tim4 in mice decreased CD301b^+^ macrophages and eventually magnified alveolar bone resorption in periodontitis. Additionally, Tim4 controlled the p38 MAPK signaling pathway to ultimately mediate CD301b^+^ macrophages phenotype. In a word, Tim4 might regulate CD301b^+^ macrophages through p38 MAPK signaling pathway in periodontitis, which provided new insights into periodontitis immunoregulation as well as help to develop innovative therapeutic targets and treatment strategies for periodontitis.

## Introduction

Periodontitis is a common chronic inflammatory disease that causes the periodontal bone destruction and may ultimately result in tooth loss.^1–3^ The World Health Organization has identified periodontitis as the dominant factor contributing to the global oral health burden and one of the top three chronic diseases.^4,5^ The pathophysiology of periodontitis involves a complex dysbiosis between pathogenic dental plaques and host immune response.^6^ Better comprehension of the regulating mechanisms of the pathogenesis may promote the development of innovative management strategies for periodontitis.

As innate immune cells, macrophages act as the main initiators of immune response^7^ and play critical roles in periodontitis.^8,9^ Their functions on inflammation and alveolar bone resorption have been given widely attentions.^10,11^ From very early on, researchers focused on the two extreme phenotypes of macrophages: proinflammatory macrophage M1 (surface marker: CD86) versus the anti-inflammatory macrophage M2 (surface marker: CD206).^12,13^ The conventional M1 and M2 phenotypes in vitro, however, are inconsistent with the heterogeneity of the macrophages in vivo according to a recent single-cell RNA sequencing (scRNA-seq) study.^14^ Besides, the classification of M1-M2 phenotype is mainly based on their inflammation modulation ability, which is insufficient to evaluate bone immunoregulation effects of macrophages. CD301b^+^ macrophages are specific to the osteoimmunology microenvironment, and are emerging as vital booster for conducting bone regeneration.^15–18^ Our recent research investigated the role of CD301b^+^ macrophages in periodontal bone remodeling.^19,20^ Interestingly, we revealed that CD301b^+^ macrophages might play an active role in orthodontic treatments under inflammatory periodontitis condition.^19^ Moreover, we reported that depletion of CD301b^+^ macrophages caused more serious bone destruction in the Mgl2-DTR mice (Mgl2, macrophage galactose N-acetyl-galactosamine specific lectin 2 gene encoding the CD301b protein) periodontitis model.^20^ Nonetheless, the key upstream targets of CD301b^+^ macrophages remain elusive to date. Thus, it is important to figure out the strategy to specifically activate CD301b^+^ macrophages, which may help to develop the new immunomodulatory therapeutic targets for periodontitis.

A newly research demonstrated that extracellular vesicles derived from human adipose-derived stem cells can enhance the expression levels of CD301b on macrophages.^21^ It has been reported that extracellular vesicles possessed a high content of phosphatidylserine (PS).^22^ Besides, Wei et al. recently revealed that the interaction of PS on extracellular vesicles with PS receptor on human umbilical cord endothelial cells contributed to the entrance of extracellular vesicles into endothelial cells to a great extent.^23^ Tim4 (T cell immunoglobulin and mucin domain-containing protein 4) is one of the best-characterized PS receptors which tethers phagocytes and apoptotic cells together during efferocytosis, enabling efficient engulfment and apoptotic cells clearance.^24–26^ Mainly existed in myeloid cells, Tim4 can reprogram macrophages to acquire an immunoregulatory, pro-resolution phenotype contributive to resolving tissue injury and boosting tissue repair.^27,28^ Macrophage-specific reduction of Tim4 expression is associated with much more serious plaque necrosis in atherosclerosis.^29^ However, the role of Tim4 in the regulation of CD301b^+^ macrophages and its potential mechanism in periodontitis are still scant.

Accordingly, it is reasonable to hypothesize that Tim4 might play a pivotal role in the regulation of CD301b^+^ macrophages concerning the underlying mechanisms between Tim4 and periodontitis. For this objective, sequencing data and a periodontitis model in Tim4-knockout mice were investigated to construct connection between Tim4 and CD301b^+^ macrophages. Selective knockdown or overexpression of Tim4 were performed to explore the potential signaling pathways.

## Results

### There exists a close connection between CD301b^+^ macrophages and Tim4 in periodontitis

**Fig. 1:**
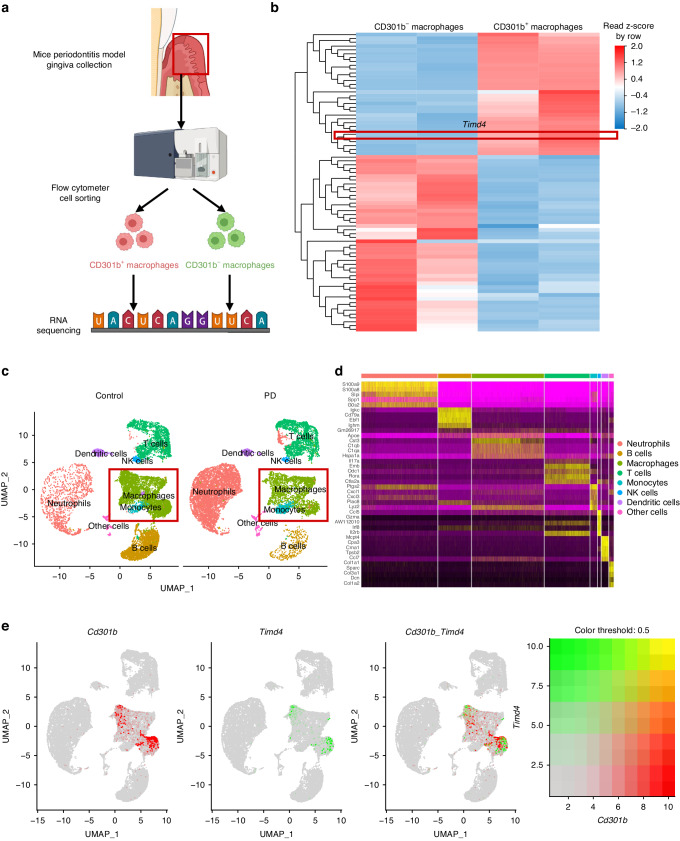
There exists a close connection between CD301b^+^ macrophages and Tim4 in periodontitis. **a** Flow chart depicting the experimental procedure for RNA sequencing assays, which was carried out on CD301b^+^ macrophages and CD301b^−^ macrophages sorted from mouse periodontal tissue by flow cytometry (Created with BioRender.com, Publication License was listed in supplemental file 2). **b** Heatmap of DEGs between CD301b^+^ macrophages and CD301b^−^ macrophages derived from RNA sequencing analysis. **c** UMAP plots of the total CD45^+^ immune cell populations identified by scRNA-seq and the major annotated cell types. **d** Heatmap illuminating the top 5 upregulated genes (ordered by decreasing *P* value) in each cluster. **e** UMAP plots showing the expression of *Cd301b* (red) and *Timd4* (green) among the total cell populations (gray). PD periodontitis

To verify whether Tim4 can serve as the key target closely associated with CD301b^+^ macrophages in periodontitis, we analyzed data (PRJNA914415) derived from high-throughput RNA sequencing which was carried out on CD301b^+^ macrophages and CD301b^−^ macrophages sorted from mouse periodontal tissue by flow cytometry in our previous study (Fig. 1a).^20^ The transcriptomes of CD301b^+^ macrophages and CD301b^−^ macrophages were compared, and significantly upregulated transcription level of *Timd4* (gene name of Tim4) in CD301b^+^ macrophages was illustrated in the heatmap (Fig. 1b). Among differentially expressed genes (DEGs), gene ontology (GO) enrichment analysis suggested that several Tim4-related functions such as apoptotic cell clearance, phagocytosis and engulfment^30^ were positively regulated by CD301b^+^ macrophages (Supplementary Fig. 1a).

To preliminarily investigate the distribution of *Cd301b* and *Timd4* gene expression among immune cell populations in periodontitis, we analyzed data (PRJNA905945) obtained from scRNA-seq which was performed on CD45^+^ immune cell populations isolated from the mouse periodontal tissue of normal and periodontitis mice (Supplementary Fig. 1b). Immune cells were profiled and UMAP method was used to illustrate major immune cell clusters (Fig. 1c, d). We further analyzed the gene expression of *Cd301b* and *Timd4* and subsequently discovered that both *Cd301b* and *Timd4* were specifically expressed in macrophages (Fig. 1e). Evidently, the gene expression of *Cd301b* and *Timd4* overlapped to large extent (Fig. 1e). Overall, these data indicated a strong association between CD301b^+^ macrophages and Tim4 in periodontitis.

### The proportions of CD301b^+^ macrophages decline during periodontitis progression and are associated with expression changes of Tim4

**Fig. 2:**
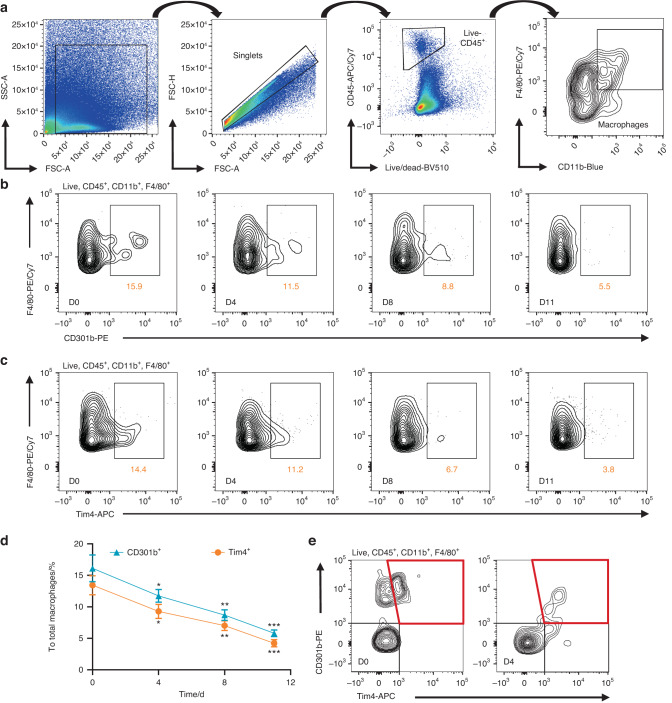
The proportions of CD301b^+^ macrophages decline during periodontitis progression and are associated with expression changes of Tim4. **a** Representative FACS pseudocolor images of gating strategy defining macrophages (CD45^+^CD11b^+^F4/80^+^ cells) from mouse periodontal tissue. Representative FACS contour plots of CD301b^+^ macrophages (**b**) and Tim4^+^ macrophages (**c**) on Day 0, 4, 8, and 11 (D0, D4, D8, and D11) after establishing a ligature-induced periodontitis model in mice. **d** The proportions of CD301b^+^ and Tim4^+^ macrophages in total macrophages on 0, 4, 8, and 11 D. *n* = 4. **e** Representative FACS contour plots describing the co-expression of CD301b and Tim4 among macrophages on Day 0 and 4 (D0 and D4). Data are depicted as line chart with mean ± SEM. *P < 0.05, **P < 0.01, and ***P < 0.001

We applied flow cytometric analysis to characterize proportions changes of CD301b^+^ macrophages and Tim4 positive expression in total macrophages (Tim4^+^ macrophages) at different time points after establishing a ligature-induced periodontitis model in mice. A six-color staining panel to gate CD45^+^CD11b^+^F4/80^+^CD301b^+^ macrophages (defined as CD301b^+^ macrophages) and CD45^+^CD11b^+^F4/80^+^Tim4^+^ macrophages (referred to Tim4^+^ macrophages) in periodontal tissue was presented in Fig. 2a~c. As the disease progressed, increasingly lower percentages of CD301b^+^ macrophages (11.5% on Day 4, *P* < 0.05; 8.8% on Day 8, *P* < 0.01; 5.5% on Day 11, *P* < 0.001) were detected compared to healthy controls (15.9% on Day 0) (Fig. 2b, d). Notably, Tim4^+^ macrophages proportions shared highly synchronized dynamic changes with CD301b^+^ macrophages proportions, as Tim4^+^ macrophages proportions gradually declined with the progression of periodontitis (14.4% on Day 0; 11.2% on Day 4, *P* < 0.05; 6.7% on Day 8, *P* < 0.01; 3.8% on Day 11, *P* < 0.001) (Fig. 2c, d). The following flow cytometric analyses confirmed that both CD301b and Tim4 were specifically expressed in macrophages (Supplementary Fig. 2a), and significant co-expression of CD301b and Tim4 was existed in macrophages (Fig. 2e). These data indicated that the proportions of CD301b^+^ macrophages were markedly decreased in the periodontitis group and closely correlated with expression changes of Tim4.

### Tim4 deficiency reduces CD301b^+^ macrophages and aggravates bone destruction in periodontitis

**Fig. 3:**
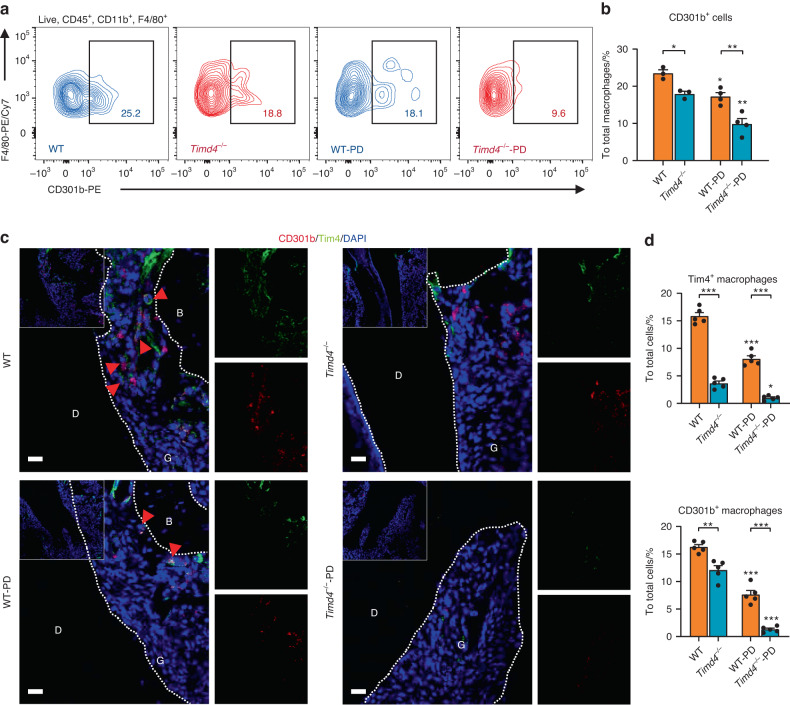
Tim4 deficiency reduces CD301b^+^ macrophages. **a** Representative FACS contour plots showing CD301b^+^ macrophages in periodontal tissue collected from *Timd4*^−/−^ mice and wild type mice with or without periodontitis. **b** The proportions of CD301b^+^ macrophages in total macrophages. **c** Immunofluorescent staining of CD301b^+^ (red) and Tim4^+^ (green) macrophages in periodontal lesions from *Timd4*^−/−^ mice and wild type mice with or without periodontitis (scale bar = 20 μm). The red arrows indicated the co-stained macrophages. **d** Quantification of Tim4^+^ and CD301b^+^ macrophages in periodontal lesions. All the periodontitis mice model was sacrificed on Day 0 and 4 after ligation. Data are depicted as bar graph with mean ± SEM. *P < 0.05, **P < 0.01, and ***P < 0.001. WT wild type mice, Timd4^−/−^ Tim4-knockout mice, WT-PD wild type mice with periodontitis, Timd4^−/−^-PD Tim4-knockout mice with periodontitis, D dentin, G gingival, B bone

**Fig. 4:**
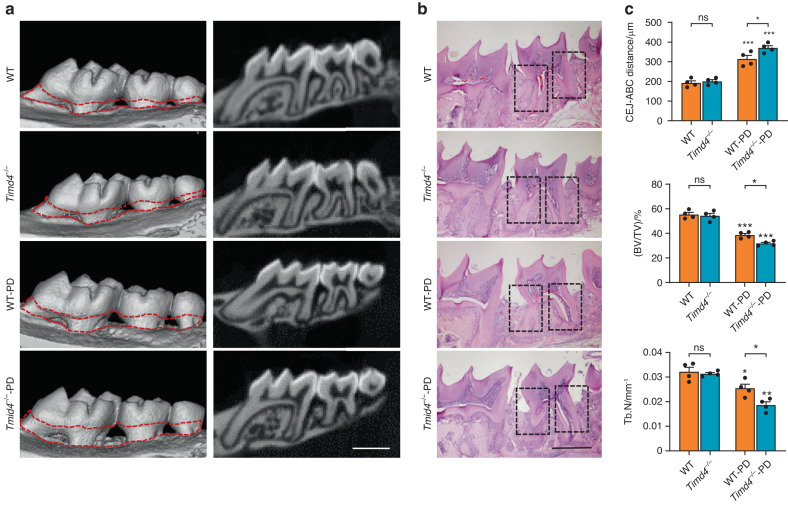
Tim4 deficiency aggravates bone destruction in periodontitis. Representative images of µCT analysis (**a**) and H&E staining (**b**) of periodontal area from *Timd4*^−/−^ mice and wild type mice with or without periodontitis. **c** Quantifications of relevant bone parameters for CEJ-ABC distance, BV/TV, and Tb.N based on three-dimensional reconstruction of the ROI in periodontal area (scale bar = 1.0 mm). All the periodontitis mice model was sacrificed on Day 0 and 4 after ligation. Data are depicted as bar graph with mean ± SEM. *P < 0.05, **P < 0.01, and ***P < 0.001. WT wild type mice, Timd4^−/−^ Tim4-knockout mice, WT-PD wild type mice with periodontitis, Timd4^−/−^-PD Tim4-knockout mice with periodontitis, CEJ-ABC cementoenamel junction to alveolar bone crest, BV/TV bone volume fraction, Tb.N trabecular bone number, ROI region of interest, ns not significant

To figure out the role of Tim4 in CD301b^+^ macrophages regulation in vivo, *Timd4*^−/−^ mice were generated and induced experimental periodontitis (Supplementary Figs. 3a~d). Staining and flow cytometric analysis of macrophages in periodontal tissue elucidated that the percentages of CD301b^+^ macrophages in *Timd4*^−/−^ mice were significantly reduced compared with wild type mice among the groups without periodontitis (Fig. 3a, b). In groups with periodontitis, same trend and more remarkable difference were observed (Fig. 3a, b). Immunofluorescence assay further validated the above results, and demonstrated that Tim4^+^ macrophages possessed the same notable trends as CD301b^+^ macrophages did (Fig. 3c, d). Additionally, sharply decreased expression levels of Tim4^+^ macrophages in *Timd4*^−/−^ mice were observed (Fig. 3c, d). In particular, overlapped expressions of CD301b and Tim4 were found (red arrows, Fig. 3c). Then, we employed µCT to analyze the bone destruction in periodontal area, and the groups with periodontitis showed significant alveolar bone resorption as compared with the groups without periodontitis, which indicated successful induction of periodontitis (red dotted area, Fig. 4a). The noticeably greater alveolar bone resorption was detected in *Timd4*^−/−^ mice as compared with wild type mice after the establishment of periodontitis (Fig. 4a, c). Further analysis of H&E staining substantiated the more serious alveolar bone resorption in *Timd4*^−/−^ mice after periodontitis induction (black dotted area, Fig. 4b). No significant difference was observed between wild type mice and *Timd4*^−/−^ mice without periodontitis (Fig. 4a~c). These findings suggested that knockout of Tim4 could reduce CD301b^+^ macrophages, followed by more serious bone destruction in periodontitis model.

### Overexpression of Tim4 promotes CD301b^+^ macrophages phenotype in vitro

**Fig. 5:**
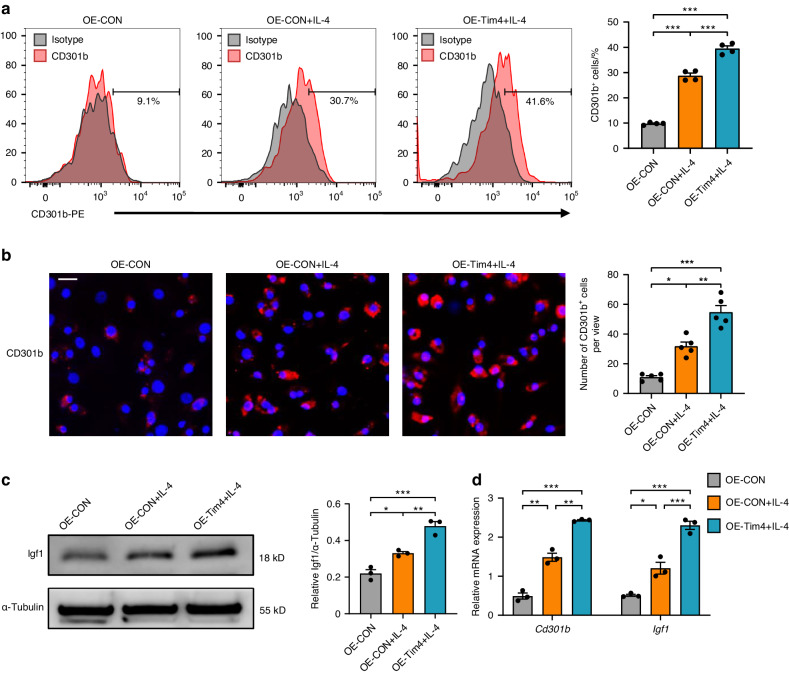
Overexpression of Tim4 promotes CD301b^+^ macrophages phenotype in vitro. **a** Representative histograms of CD301b-PE stained for flow cytometry. The percentages of CD301b^+^ cells in total macrophages were determined. **b** Immunofluorescent staining of CD301b in BMDMs (scale bar = 20 μm). The numbers of CD301b^+^ cells were statistically determined. **c** The expression levels of Igf1 were detected by Western blot. **d** The expression levels of *Cd301b* and *Igf1* were observed by RT-qPCR. Data are depicted as bar graph with mean ± SEM. *P < 0.05, **P < 0.01, and ***P < 0.001. OE-Tim4 Tim4 overexpressed BMDMs, OE-CON control overexpressed BMDMs, Igf1 insulin-like growth factor 1

Tim4 was overexpressed via a lentivirus-carrying Tim4 expression cassette. Successful transfection was confirmed via Western blot, flow cytometry, and RT-qPCR (*P* < 0.001; Supplementary Fig. 4). Flow cytometry and immunofluorescence of cells exhibited higher amounts of CD301b^+^ macrophages after exposed to IL-4 for 24 h (Fig. 5a, b), validating our previous inducing strategy.^20^ Tim4 overexpression further markedly increased the percentages of CD301b^+^ macrophages (Fig. 5a, b).

Insulin-like growth factor 1 (Igf1) was one of the most essential growth factors for bone remodeling and has been described as the representative functional molecule of CD301b^+^ macrophages.^20,31^ Accordingly, we next explored whether the overexpression of Tim4 altered Igf1 protein level utilizing Western blot. The expression of Igf1 was upregulated via IL-4 stimulation, which was further enhanced after Tim4 overexpression (Fig. 5c). Moreover, RT-qPCR results showed that the transcription levels of *Cd301b* and *Igf1* were also augmented in response to combined Tim4 overexpression and IL-4 treatment, compared to the treatment with IL-4 alone (Fig. 5d). Collectively, the above data implied that overexpression of Tim4 could facilitate CD301b^+^ macrophages phenotype.

### Knockdown of Tim4 inhibits CD301b^+^ macrophages phenotype in vitro

**Fig. 6:**
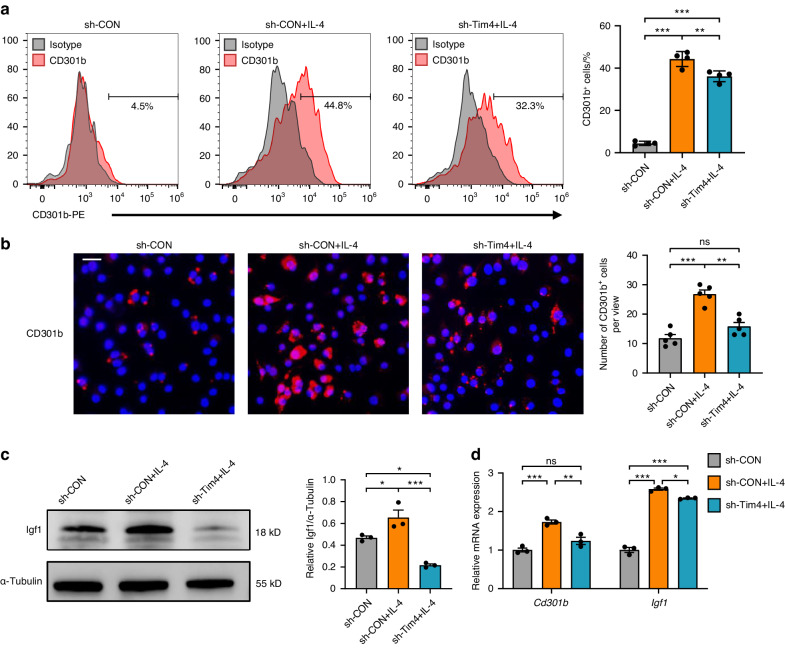
Knockdown of Tim4 inhibits CD301b^+^ macrophages phenotype in vitro. **a** Representative histograms of CD301b-PE stained for flow cytometry. The percentages of CD301b^+^ cells in total macrophages were determined. **b** Immunofluorescent staining of CD301b in BMDMs (scale bar = 20 μm). The numbers of CD301b^+^ cells were statistically determined. **c** The expression levels of Igf1 were detected by Western blot. **d** The expression levels of *Cd301b* and *Igf1* were observed by RT-qPCR. Data are depicted as bar graph with mean ± SEM. *P < 0.05, **P < 0.01, and ***P < 0.001. sh-Tim4 Tim4 knocked down BMDMs, sh-CON control knocked down BMDMs, ns not significant, Igf1 insulin-like growth factor 1

Tim4 was knocked down via specific small hairpin RNA (shRNA), and its efficiency has been examined (*P* < 0.001; Supplementary Fig. 5). After IL-4 induction, higher percentages of CD301b^+^ macrophages were observed by flow cytometry and immunofluorescence assays, while the selective knockdown of Tim4 could remarkably reduce the percentages of CD301b^+^ macrophages (Fig. 6a, b).

In addition, Western blot revealed an elevated expression of Igf1 after IL-4 stimulation compared with control, which was decreased after Tim4 inhibition (Fig. 6c). RT-qPCR results further verified that mRNA expression levels of *Cd301b* and *Igf1* were also attenuated after Tim4 inhibition (Fig. 6d). Therefore, the above data demonstrated the inhibition effect of Tim4 knockdown on CD301b^+^ macrophages phenotype.

### Tim4 mediates CD301b^+^ macrophages phenotype via p38 MAPK signaling pathway in vitro

**Fig. 7:**
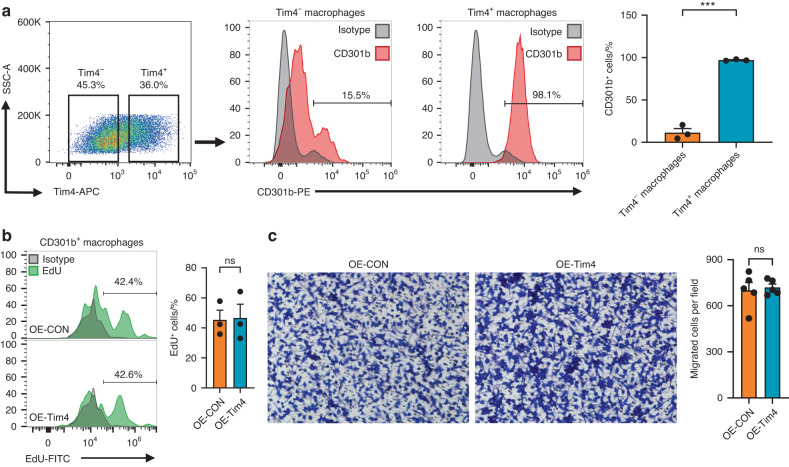
Exploration of the underlying mechanisms of Tim4-mediated CD301b^+^ macrophages phenotype. **a** Representative FACS plots of Tim4-APC and CD301b-PE stained for flow cytometry. The percentages of CD301b^+^ macrophages in Tim4^−^ or Tim4^+^ macrophages from Tim4 overexpressed BMDMs were determined. **b** Representative histograms of EdU-FITC stained for flow cytometry performed by EdU assay. The percentages of EdU^+^ cells in CD301b^+^ macrophages from OE-CON or OE-Tim4 were determined. **c** CD301b^+^ macrophages passing through the filters on the bottom of the membrane were stained with crystal violet for transwell assay (magnification, ×20). The numbers of CD301b^+^ macrophages recruited by the OE-CON or OE-Tim4 culture supernatants were statistically determined. Data are depicted as bar graph with mean ± SEM. ***P < 0.001. OE-CON control overexpressed BMDMs, OE-Tim4 Tim4 overexpressed BMDMs, EdU 5-Ethynyl-2’-deoxyuridine

To find out the underlying mechanisms, we first applied flow cytometry and found a highly abundant of CD301b^+^ macrophages among Tim4^+^ macrophages compared to Tim4^−^ macrophages after Tim4 overexpression, revealing the mode of action of Tim4 termed as “cell-autonomous” (Fig. 7a). Cell-autonomous mechanism was described as a differentiation process regulated by transcriptional levels of genes within the cell,^32^ indicating that the role of Tim4 rather than the whole cell populations (containing both Tim4^−^ macrophages and Tim4^+^ macrophages) was responsible for modulating phenotypic differentiation of macrophages into CD301b^+^ macrophages (Fig. 7a). EdU assay and transwell assay were performed to further exclude the pro-proliferative and pro-migratory effects of Tim4 on CD301b^+^ macrophages, as no significant difference was observed between OE-CON (control overexpressed BMDMs) group and OE-Tim4 (Tim4 overexpressed BMDMs) group (Fig. 7b, c).

**Fig. 8:**
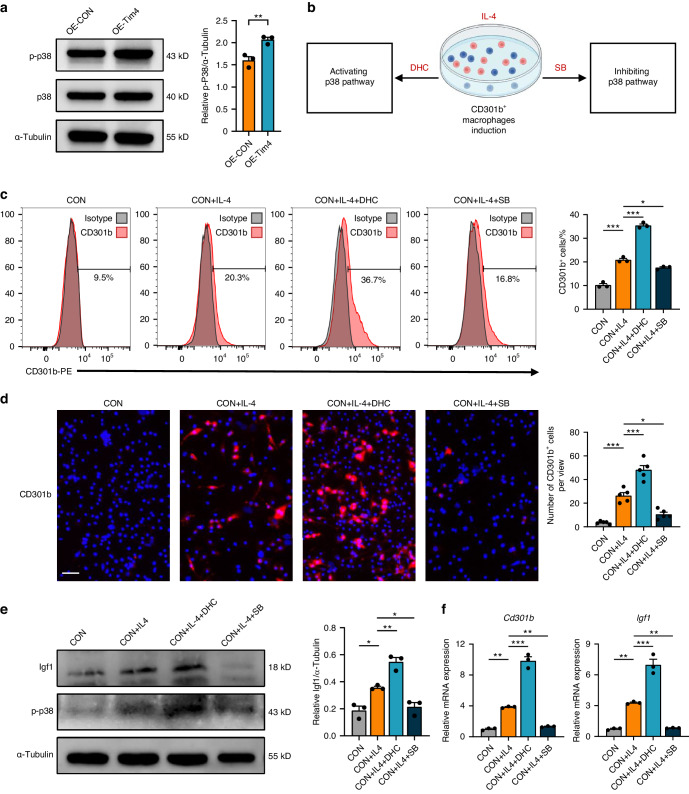
Tim4 mediates CD301b^+^ macrophages phenotype via p38 MAPK signaling pathway in vitro. **a** The expression levels of the key molecules of p38 MAPK signaling pathway in OE-CON or OE-Tim4 were detected by Western blot. **b** Schematic representation of the experimental procedure (Created with BioRender.com, Publication License was listed in supplemental file 5). Mice BMDM were induced to be CD301b^+^ macrophages with IL-4 stimulation and then subjected to p38 pathway activation or inhibition. **c** Representative histograms of CD301b-PE stained for flow cytometry. The percentages of CD301b^+^ cells in total macrophages from groups with different treatments were determined. **d** Immunofluorescent staining of CD301b in BMDMs (scale bar = 50 μm). The numbers of CD301b^+^ cells in groups with different treatments were statistically determined. **e** The expression levels of Igf1 in groups with different treatments were detected by Western blot. **f** The expression levels of *Cd301b* and *Igf1* in groups with different treatments were observed by RT-qPCR. Data are depicted as bar graph with mean ± SEM. *P < 0.05, **P < 0.01, and ***P < 0.001. OE-CON control overexpressed BMDMs, OE-Tim4 Tim4 overexpressed BMDMs, IL-4 interleukin 4, DHC dehydrocorydaline (specific agonist for the p38 pathway), SB SB203580 (specific inhibitor for the p38 pathway), CON control BMDMs, Igf1 insulin-like growth factor 1

**Fig. 9:**
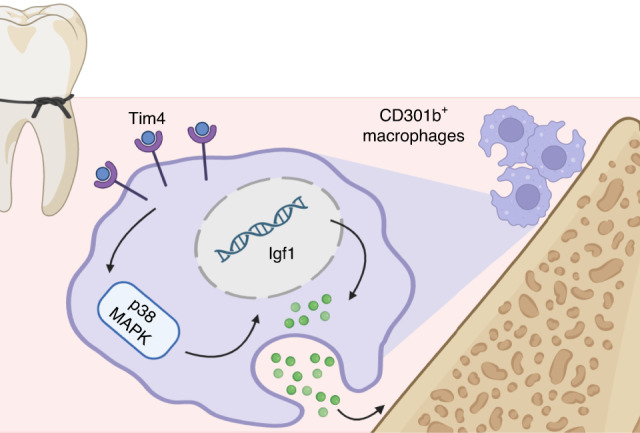
Schematic diagram of the mechanism (Created with BioRender.com, Publication License was listed in supplemental file 6). Tim4 might regulate CD301b^+^ macrophages phenotype through p38 MAPK signaling pathway in periodontitis

Evidences have described that Tim4 in macrophages could mediate p38 MAPK signaling pathway.^33,34^ Qin et al. demonstrated that Tim4 contributed to the epithelial to mesenchymal transition process and promoted nasal polyp formation via the ROS/p38 MAPK/Egr-1 pathway.^34^ Yeung et al. reported that inhibition of Tim4 expression reduced the expression of inflammatory cytokines through the p38 MAPK signaling pathways.^35^ Thus, p38 MAPK signaling pathway was taken into consideration to investigate the signal pathways of Tim4 in CD301b^+^ macrophages phenotype regulation. Western blot showed that Tim4 overexpression significantly increased the phosphorylation of p38 MAPK (Fig. 8a). To confirm the role of p38 pathway in Tim4-mediated CD301b^+^ macrophages phenotype, we activated or blocked p38 pathway with specific chemical agonist Dehydrocorydaline (DHC) or inhibitor SB203580 (Fig. 8b). Flow cytometry and immunofluorescence staining showed that activating p38 pathway notably increased CD301b^+^ macrophages, whereas inhibiting p38 pathway largely decreased CD301b^+^ macrophages (Fig. 8c, d). Western blot and RT-qPCR results demonstrated highly consistent expression changes of Igf1 with that of CD301b^+^ macrophages (Fig. 8e, f). Taken together, as shown in Fig. 9, the schematic diagram illustrated that Tim4 might regulate CD301b^+^ macrophages phenotype through p38 MAPK signaling pathway in periodontitis.

## Discussion

Exploration of the immunoregulation mechanisms of the pathogenesis is critically important for periodontitis management. CD301b^+^ macrophages, distinct from traditional M1 and M2 macrophages, are emerging as a crucial macrophage subpopulation with notable bone immunomodulatory functions.^15–20,36^ However, the central upstream targets and concerning mechanisms in the regulation of CD301b^+^ macrophages in periodontitis remain poorly investigated. Here, we concentrated on the role of Tim4, a latent upstream regulator of CD301b^+^ macrophages. We first demonstrated that Tim4 was strongly associated with CD301b^+^ macrophages during periodontitis progression. Furthermore, the deficiency of Tim4 in mice decreased CD301b^+^ macrophages and eventually magnified alveolar bone resorption in periodontitis. Additionally, Tim4 controlled the p38 MAPK signaling pathway to ultimately mediate CD301b^+^ macrophages phenotype.

CD301b^+^ macrophages have been recently identified as a group of macrophages focused on regulating the immune response and regeneration functions. Sommerfeld et al. demonstrated for the first time that macrophages could be divided by the surface marker CD301b, and CD301b^+^ macrophages were specific to the regenerative microenvironment, rather than CD206^+^ macrophages (M2-macrophage) or CD86^+^ macrophages (M1-macrophage).^17^ In another recent research, transplantation with CD301b^+^/CD206^+^ macrophages achieved better skin regeneration compared with CD301b^−^/CD206^+^ macrophages.^15,16^ Notably, CD301b^+^ macrophage was not a subpopulation of M2 macrophages, as CD301b^+^ macrophages possessed both M1 functional factors such as IL1 and M2 functional cytokine genes such as Arg1, which implied a more comprehensive role in conducting tissue regeneration.^18^ Our recent study found that there existed strong correlations between the dynamic changes in CD301b^+^ macrophages and CD206^+^ macrophages during periodontitis progression, however, the expression pattern and localization of CD301b^+^ macrophages were largely different from those of CD206^+^ macrophages in periodontal tissue.^20^ Considering the specificity of CD301b^+^ macrophage phenotype, it is reasonable to explore the strategy to specifically induce CD301b^+^ macrophages.

Inspiringly, a recent study has discovered a novel inducer of CD301b^+^ macrophages, and it reported that extracellular vesicles could incredibly induce the overexpression of CD301b on macrophages.^21^ High content of PS has been found in extracellular vesicles.^22^ Besides, another research demonstrated that the interaction of PS on extracellular vesicles with PS receptor on target cells played a central part when extracellular vesicles entered into target cells.^23^ Tim4, known as one of the best-characterized PS receptors, was closely involved in the process of efficient engulfment and apoptotic cells clearance via tethering phagocytes and apoptotic cells together during efferocytosis.^26^ In this study, the transcriptome sequencing analysis identified a high level of *Timd4* expression on CD301b^+^ macrophages in periodontal tissue. In addition, GO enrichment analysis revealed that the processes of apoptotic cell clearance, phagocytosis, and engulfment were positively regulated by CD301b^+^ macrophages. Moreover, the following gene knockout mice experiment exhibited that deficiency of Tim4 significantly reduced CD301b^+^ macrophages in periodontal tissue. Consequently, it is possible that Tim4 might be engaged in the regulation of CD301b^+^ macrophages in periodontitis.

The earliest research on CD301b^+^ macrophages was based on the environment of adipose tissue.^37,38^ Studies have shown that the adipose-tissue-resident macrophage population was enriched for CD301b marker, and adipose tissue could provide a niche for CD301b^+^ macrophages.^39^ Coincidentally, increasing data have demonstrated that Tim4 was defined as representative cell surface marker for resident adipose tissue macrophages, displaying essential role in modulation of adipose tissue homeostasis.^39–43^ Here, we attempted to locate *Cd301b* and *Timd4* in periodontal tissue through scRNA-seq and flow cytometry technologies. Both specifically expressed in macrophages, *Cd301b* and *Timd4* displayed largely overlapped expression patterns, indicating that Tim4 might serve as a promising target of CD301b^+^ macrophages in periodontitis.

Interestingly, the current study found that with the progression of periodontitis, the expression of Tim4 on macrophages in periodontal tissue became increasingly scarce, which shared the same dynamic changes with the proportions of CD301b^+^ macrophages. After generating gene knockout mice, deficiency of Tim4 brought about much less CD301b^+^ macrophages and intensified periodontitis, resulting in greater alveolar bone resorption. Accumulating evidence has described that CD301b^+^ macrophages play a leading role in prompting fibroblast proliferation, adipocyte proliferation, osteogenesis and angiogenesis, and CD301b^+^ macrophages have been recognized as an advanced tissue regenerative macrophage subpopulation.^15,16,31,36^ In our recent work, Wang et al. found that depletion of CD301b^+^ macrophages caused more serious bone destruction in CD301b-deficient mice periodontitis model.^20^ Comparatively speaking, Tim4 can reprogram macrophages phenotype to trigger immunoregulatory and pro-resolution functions, followed by resolving tissue injury and boosting tissue repair.^27^ It is also noteworthy that Hoeffel et al. recently revealed in a skin damage mice model, the neuropeptide TAFA4 produced in skin ensured the survival and maintenance of IL-10^+^Tim4^+^ dermal macrophages, reducing skin damage and promoting tissue regeneration.^44^ Overall, we suggested that declined Tim4 expression, which was closely associated with the decreased CD301b^+^ macrophages, might contribute to the pathogenetic process of periodontitis.

Several studies revealed that IL-4 receptor subunit alpha (IL-4Rα) was highly expressed on CD301b^+^ macrophages and IL-4 was considered as a kind of stimulant for CD301b^+^ macrophages induction.^17,20,45^ In this study, in vitro experiments showed that IL-4 stimulation dramatically elevated CD301b^+^ macrophages amount, aligned to the above findings. Despite efficient induction of CD301b expression, IL-4 is clearly not unique to CD301b^+^ macrophages, as it acts extensively on various cells including B cells, T cells, mast cells, macrophages, and so on.^46^ Besides, the regulating effects of IL-4 on M2 macrophages have been well delineated,^47^ which may consequently influence the functions of CD301b^+^ macrophages in in vivo application.^20^ The present data of increased or decreased CD301b^+^ macrophages resulting from overexpression or knockdown of Tim4 might address the dilemma. On the one hand, in vitro experiments confirmed that Tim4 functioned as a positive regulator of CD301b^+^ macrophages. On the other hand, Tim4 was presented as a much more specific activator than IL-4.

Currently, it is challenging to accurately orchestrate the immune responses to improve osteogenesis in bone regeneration.^48–50^ Igf1 is a hormone with known actions on bone growth and has been reported to be highly expressed by CD301b^+^ macrophages.^15,16,51^ It has been validated that CD301b^+^ macrophage played an important role in promoting osteogenic differentiation of BMSCs by secretion of Igf1.^20,36^ A requisite role for CD301b^+^ macrophage-derived Igf1 in regulating bone formation has been well documented in periodontitis, as CD301b^+^ macrophages in periodontal tissue displayed several osteogenesis-related processes and expressed high level of related gene Igf1.^20^ Western blot and RT-qPCR analyses demonstrated that Tim4 overexpression or knockdown could enhance or attenuate the expression of Igf1, which supported above observations. Based on our findings, we speculated that Tim4 was capable of positively regulating CD301b^+^ macrophages phenotype, and might sever as a bone repair-promoting factor from therapeutic perspective which could improve the development of periodontitis management.

p38 MAPK signaling pathway is essential for bone formation and bone strength in vivo.^52,53^ The p38 MAPK pathway also plays a central role in the regulation of monocyte/macrophage development and differentiation.^54–57^ To validate the mechanism of Tim4-regulated CD301b^+^ macrophages phenotype, we focused on p38 MAPK signaling pathway because evidences have described that Tim4 in macrophages could mediate p38 MAPK signaling pathway and might facilitate tissue regeneration.^34,35^ The results indicate that Tim4-mediated p38 MAPK signaling pathway was responsible for modulating phenotypic differentiation of macrophages into CD301b^+^ macrophages.

Herein, we considered Tim4, the typical PS receptor and newly marker for tissue resident macrophages,^41^ as a potential target of CD301b^+^ macrophages in periodontal tissue. This study not only provides evidence for the key role of Tim4 for the upstream regulation of CD301b^+^ macrophages, but also implies the origin of CD301b^+^ macrophages. There is a limitation in the study regarding the lack of in vitro functional verifications for Tim4, which are needed in the future research on the underlying mechanisms between Tim4 and periodontitis.

In conclusion, our study was the first to demonstrated the crucial upstream target of CD301b^+^ macrophages, showing that Tim4 might regulate CD301b^+^ macrophages phenotype in periodontitis through p38 MAPK signaling pathway. Of note, Tim4 deficiency decreased CD301b^+^ macrophages and exacerbated alveolar bone destruction in mice periodontitis model, which provided new insights into periodontitis immunoregulation as well as help to develop innovative therapeutic targets and treatment strategies for periodontitis.

## Materials and methods

### Data collection and processing

Publicly available RNA sequencing datasets were obtained from Sequence Read Archive DataSets PRJNA914415. For all upregulated and downregulated DEGs, GO enrichment analysis and heatmap analysis were processed with the free online Dr. TOM II Platform. For scRNA-seq datasets PRJNA905945, the downstream analysis steps were performed using the R v3.5.1 software and Seurat package. Next, for quality control of each matrix, living periodontal cells defined by using the percentage of mitochondrial gene expression as an inclusion criterion (<10%) were retained, and cells with genes <200 or >5 000 were filtered out. The matrix was normalized for sequencing depth using the “NormalizeData” function. The top 2 000 variable genes of each matrix were detected applying principal component analysis. For the clustering of the whole cells, the resolution value was set to 0.8 and the integrated data were dimension reduced with pc use = 1:10. The cell clusters were annotated based on MouseRNAseqData dataset. The visualization of UMAP, heatmap, and featurePlot is done when invoking the “DimPlot”, “Doheatmap” and “FeaturePlot” functions respectively.

### Ethical approval and mice model

Female C57BL/6J mice aged 8 weeks were provided by Vital River Laboratory Animal Technology Co. (Beijing, China). Tim4-knockout (*Timd4*^−/−^) mice on the C57BL/6 background were purchased from Model Organisms Center, Inc. (Shanghai, China). Mice were maintained under SPF conditions and housed in the specific pathogen-free, temperature-controlled facilities. All animal studies were performed with the approval of the Animal Care and Use Committee of the Medical Research Institute, Wuhan University (MLIC2021175) and in accordance with the ARRIVE guidelines 2.0.

The ligature-induced periodontitis model was established by tying 5-0 silk ligatures around the left and right maxillary second molars as previously described,^58^ and then sacrificed at different time points after ligation (specified in the figure legends).

### Flow cytometry

To obtain single-cell suspensions, the periodontal soft tissues surrounding the buccal and lingual sides of the mouse maxillary molars were excised, minced and collected. The samples were then digested with 2 ml RPMI-1640 medium (HyClone, Logan, UT, USA) containing 10% fetal bovine serum (FBS, Gibco, New York, NY, USA), collagenase type II (2 mg/ml, Thermo Fisher Scientific, CA, USA) and collagenase type IV (2 mg/ml, Thermo Fisher Scientific) for 2 h at 37°C in a shaker bath. Subsequently, the digested tissues were ground and strained with 70-μm filters (Thermo Fisher Scientific) under PBS washing, and the filtrates were centrifuged with a speed of 2 500 r/min for 5 min.

Freshly isolated single cells were suspended in PBS and were first stained with Fixable Viability Stain 510 (FVS510, 1:1 000, BD Biosciences, USA) for 20 min to determine living cells. Anti-CD16/32 antibody (1:100, Biolegend, San Diego, CA, USA) was applied to block non-specific binding of immunoglobulin to the Fc receptors before surface dyeing. The fluorochrome-conjugated antibodies of surface markers were listed in Table 1. After 30 min of incubation in the dark at 4 °C, cells were resuspended in 200 μL PBS. Flow cytometry was conducted on an LSR FortessaX20 (BD Biosciences, USA) and the data analysis was performed on the software FlowJo 10.4 (FlowJo LLC, Ashland, OR, USA).

**Table 1:** Description of the antibodies used for flow cytometry

Marker	Label	Source	Clone	Dilution
CD16/CD32	(None)	Biolegend	93	1: 100
FVS510	BV510	BD	(None)	1: 1 000
CD45	APC-Cy7	Biolegend	30-F11	1: 400
CD11b	Pacific Blue	Biolegend	M1/70	1: 200
F4/80	PE-Cy7	Biolegend	BM8	1: 800
CD301b	PE	Biolegend	URA-1	1: 200
Tim4	APC	Biolegend	RMT4-54	1: 200

### Histological staining

Fixed and decalcified mouse maxillae were dehydrated in 30% sucrose solution for 48 h at 4 °C and embedded in optimum cutting temperature (OCT, Sakura, America), followed by cryosection which cut samples into 8 μm thickness sections. For immunofluorescence, the sections were stained with primary antibodies against CD301b (1: 100, Invitrogen) together with Tim4 (1: 100, Sigma-Aldrich, USA), and the secondary antibodies with 488 and 593 fluorescence markers (1: 200, Invitrogen) were adopted. Images were harvested using the laser scanning confocal microscope (LSCM, Leica, Germany). The histomorphometric analysis was performed by ImageJ software (National Institutes of Health, Bethesda, MD, USA). The sections were then stained with hematoxylin and eosin (H&E, Google biotechnology, China) according to the manufacturer’s instructions.

### Micro-computed tomography

Fixed mouse maxillary bones were assessed for bone parameters applying a micro-computed tomography (µCT, Skyscan1276, Bruker) system and affiliated analyzing software. The scans were performed at 55 kV and 200 mA with a resolution of 10 µm. The distance between the cementoenamel junction and alveolar bone crest (CEJ-ABC) of the maxillary second molar on the distal side was measured by Data Viewer program. The area of interradicular alveolar bone of the second molar was defined as the region of interest (ROI). The bone loss in the ROI was calculated by CTAn program for quantification, including the bone volume fraction (BV/TV) and trabecular bone number (Tb.N).

### Isolation and culture of bone marrow-derived macrophages (BMDMs)

Bone marrow cells were extracted from the tibias and femurs of mice and cultured with high glucose Dulbecco’s modified eagle’s medium (DMEM, Hyclone, USA), in the presence of 20% FBS, 1% penicillin–streptomycin (Hyclone), and macrophage colony stimulating factor (M-CSF, 20 ng/mL, PeproTech, USA). Five days later, the mature BMDMs were eligible for further experiments.

For overexpression or knockdown of Tim4, lentivirus-carrying mouse Tim4 expression cassette or Tim4 small hairpin RNA (shRNA) lentiviral vector was designed and transfected into BMDMs, respectively. An empty lentivirus was applied as control (OE-CON or sh-CON). After transfection, BMDMs were screened with puromycin and termed as OE-Tim4 or sh-Tim4. The transfection procedures were performed according to the protocol of the transfection reagent (PT-114-15, jetPrime, France). To induce CD301b^+^ macrophages in vitro, BMDMs were treated with interleukin (IL)-4 (20 ng/mL, Biolegend) for 24 h.^20^ To verify the signaling pathway involved, specific chemical agonist Dehydrocorydaline (DHC, 20 μmol/L, MCE) and inhibitor SB203580 (SB, 10 μmol/L, Selleck) for the p38 MAPK pathway were added into the culture medium and activated for 48 h.

### Real-time quantitative PCR (RT-qPCR)

Total RNA was isolated via Trizol reagent (Invitrogen) from cells or tissues in accordance with the manufacturer’s instructions (Takara, Shiga, Japan). The RNA was reverse-transcribed into cDNA using PrimeScript RT Master Mix (Takara, Japan). RT-qPCR was carried out with a LightCycler 96 System (Roche, Basal, Switzerland) applying SYBR Green reagents (Takara). Relative gene expression levels normalized to *Gapdh* were determined and the data were analyzed using the 2-ΔΔct method. The primers designed for target genes were shown in Table 2.

**Table 2:** Primers designed for qRT-PCR

Gene	Forward primer	Reverse primer
*Gapdh*	GACTGATGTTGTTGACAGCCACTG	TAGCCACTCCTTCTGTGACTCTAAC
*Cd301b*	TTAGCCAATGTGCTTAGCTGG	GGCCTCCAATTCTTGAAACCT
*Timd4*	GTGTACTGCTGCCGTATAGAGG	TGGTGGTTGGGAGAACAGATG
*Igf1*	TAGCCACTCCTTCTGTGACTCTAAC	TAGCCACTCCTTCTGTGACTCTAAC

### Western blot

Mouse periodontal soft tissues and BMDMs were homogenized manually and lysed in radio immunoprecipitation assay (RIPA) buffer (Beyotime Biotechnology, China) containing protease inhibitor and phosphatase inhibitor. All samples were quantified and normalized by bicinchoninic acid (BCA) kit (Thermo Fisher Scientific). Protein extracts were loaded into sodium dodecyl sulfate polyacrylamide gel electrophoresis (SDS-PAGE) and transferred to polyvinylidene fluoride (PVDF, Millipore, USA) membranes, which were then blocked in 5% skim milk for 1 h at room temperature (RT). The membranes were incubated with primary antibodies against Tim4 (1:1 000, Sigma-Aldrich), Igf1 (1:1 000, ABclonal, China), p-p38 (1:1 000, CST, USA), p38 MAPK (1:1 000, CST), and α-Tubulin (1: 5 000, ABclonal) overnight at 4 °C, followed by anti-rabbit horseradish peroxidase (HRP)-conjugated secondary antibody (1: 10 000, Proteintech, China, 1:5 000) for 1 h at RT. The results were visualized with a chemiluminescence imaging system and further analyzed by ImageJ software. Relative protein expression levels normalized to α-Tubulin were evaluated.

### 5-Ethynyl-2′-deoxyuridine (EdU) cell proliferation assay

The EdU-488 Cell Proliferation Kit (Beyotime) was employed to detect the effect of macrophages proliferation. Briefly, a density of 1 × 10^4^ treated BMDMs were seeded in 24-well plates in the presence of DMEM medium containing 10% FBS and 10 μmol/L EdU. After 2 h, the isolated cells were fixed with 4% paraformaldehyde (PFA) for 30 min at RT. Click Additive Solution (with Azide 488) was prepared and incubated with cells for 30 min in the dark at 4 °C, followed by PBS washing. The fluorescent-labeled cells can be visualized under the flow cytometer and the cell proliferation can be determined.

### Transwell assay

The migration assay was performed using a 24-well transwell chamber with 5 μm pore inserts (Corning, USA). The bottom of the lower chamber was filled with 500 μl culture supernatants of OE-CON/OE-Tim4 as a chemoattractant, while the CD301b^+^ macrophages (5 × 10^4^ cells per well) obtained by sorting BMDMs were suspended in serum-free medium and plated in the upper chamber. The established culture system was incubated for 48 h at 5% CO_2_ and 37 °C. After incubation, the remaining cells on the top of the membrane were wiped off and the cells through the filters on the bottom of the membrane were fixed in 4% PFA for 20 min. The inserts were stained with 1% crystal violet for 10 min. The migration results were quantified using ImageJ.

### Statistical analysis

Statistical analysis was performed with GraphPad Prism 8.0 (San Diego, CA, USA) software. The differences among groups were investigated through Student’s t test and one-way analysis of variance (ANOVA). All experiments were repeated at least 3 times and the numerical data were displayed as mean ± SEM, and the statistically significance level was set at *P* < 0.05.

### Supplementary information

Supplemental file 1-Revised Supplementary Figure

Supplemental file 2-Publication License

Supplemental file 3-Publication License

Supplemental file 4-Publication License

Supplemental file 5-Publication License

Supplemental file 6-Publication License

## Data Availability

The data used and/or analyzed during the current study are contained within the manuscript. RNA sequencing data are available at the Sequence Read Archive database under the accession number PRJNA914415. Single-cell RNA sequencing data are available at the Sequence Read Archive^59^ database under the accession number PRJNA 863330. Other data are available from the corresponding author on reasonable request.
