# Supplementary material for: Tim4 deficiency reduces CD301b+ macrophage and aggravates periodontitis bone loss
Source: Int J Oral Sci. 2024 Feb 28;16:20. doi: 10.1038/s41368-023-00270-z (PMC10902347; doi:10.1038/s41368-023-00270-z)
Supplement: Supplementary file 1 — Supplemental file 1-Revised Supplementary Figure [file 41368_2023_270_MOESM1_ESM.docx]

Tim4 deficiency reduces CD301b^+^ macrophage and aggravates periodontitis bone loss

Ziming Wang^1†^, Hao Zeng^1†^, Can Wang^1^, Jiaolong Wang^4^, Jing Zhang^1^, Shuyuan Qu^1^, Yue Han^1^, Liu Yang^1^, Yueqi Ni^1^, Wenan Peng^1^, Huan Liu^1^, Hua Tang^3^, Qin Zhao^1*^, Yufeng Zhang^1,2*^

^1^State Key Laboratory of Oral & Maxillofacial Reconstruction and Regeneration, Key Laboratory of Oral Biomedicine Ministry of Education, Hubei Key Laboratory of Stomatology, School & Hospital of Stomatology, Taikang Center for Life and Medical Sciences, Wuhan University, Wuhan 430079, China

^2^Medical Research Institute, School of Medicine, Wuhan University, Wuhan 430071, China

^3^Institute of Infection and Immunity, Science and Technology Innovation Center, Shandong First Medical University & Shandong Academy of Medical Sciences, Jinan, 250000, Shandong, PR China

^4^School of Stomatology, Nanchang University, Nanchang 330006, China

^†^Authors contributing equally to this article

**^*^Corresponding Author:**

State Key Laboratory of Oral & Maxillofacial Reconstruction and Regeneration, Key Laboratory of Oral Biomedicine Ministry of Education, Hubei Key Laboratory of Stomatology, School & Hospital of Stomatology, Taikang Center for Life and Medical Sciences, Wuhan University, Wuhan 430079, China

Qin Zhao, Email: zhaoqin@whu.edu.cn

Yufeng Zhang, Email: zyf@whu.edu.cn

Running title

Tim4 regulates CD301b^+^ macrophage in periodontitis

**Supplementary Figure legends**

**Supplementary Figure 1. a** GO enrichment analysis of DEGs between CD301b^+^ macrophages and CD301b^−^ macrophages derived from RNA sequencing assays. GO, gene ontology. **b** Flow chart depicting the experimental procedure for scRNA-seq assays, which was performed on CD45^+^ immune cell populations isolated from the mouse periodontal tissue of normal and periodontitis mice (Created with BioRender.com, Publication License was listed in supplemental file 3). scRNA-seq, single-cell RNA sequencing.

**Supplementary Figure 2. a** Representative FACS plots respectively depicting the co-expression of CD301b or Tim4 with F4/80 among CD45^+^CD11b^+^ cells.

**Supplementary Figure 3.** Gene identification of *Timd4*^−/−^ mice. **a** Genotyping by PCR. RT-qPCR (**b**), WB (**c**), and Flow cytometry (**d**) were used to detect the expression of Tim4 in periodontal tissue. Data are depicted as bar graph with mean ± SEM. ***P* < 0.01, and ****P* < 0.001. WT, wild type mice; +/−, Tim4 heterozygous mice; −/−, Tim4-knockout mice; *Timd4*^−/−^, Tim4-knockout mice.

**Supplementary Figure 4.** **a** Schematic representation of the experimental procedure (Created with BioRender.com, Publication License was listed in supplemental file 4). Mice BMDM were induced to be CD301b^+^ macrophages with IL-4 stimulation and then subjected to Tim4 overexpression. Tim4 over-expression efficiency was examined via Western blot (**b**), flow cytometry (**c**), and RT-qPCR (**d**). Data are depicted as bar graph with mean ± SEM. ****P* < 0.001. BMDM, bone marrow-derived macrophages; Con, control; IL-4, interleukin 4; M-CSF, macrophage colony stimulating factor; OE-CON, control overexpressed BMDMs; OE-Tim4, Tim4 overexpressed BMDMs.

**Supplementary Figure 5.** **a** Schematic representation of the experimental procedure (Created with BioRender.com, Publication License was listed in supplemental file 4). Mice BMDM were induced to be CD301b^+^ macrophages with IL-4 stimulation and then subjected to Tim4 knockdown. Tim4 knock-down efficiency was examined via Western blot (**b**), flow cytometry (**c**), and RT-qPCR (**d**). Data are depicted as bar graph with mean ± SEM. ****P* < 0.001. BMDM, bone marrow-derived macrophages; Con, control; IL-4, interleukin 4; M-CSF, macrophage colony stimulating factor; sh-CON, control knocked down BMDMs; sh-Tim4, Tim4 knocked down BMDMs.


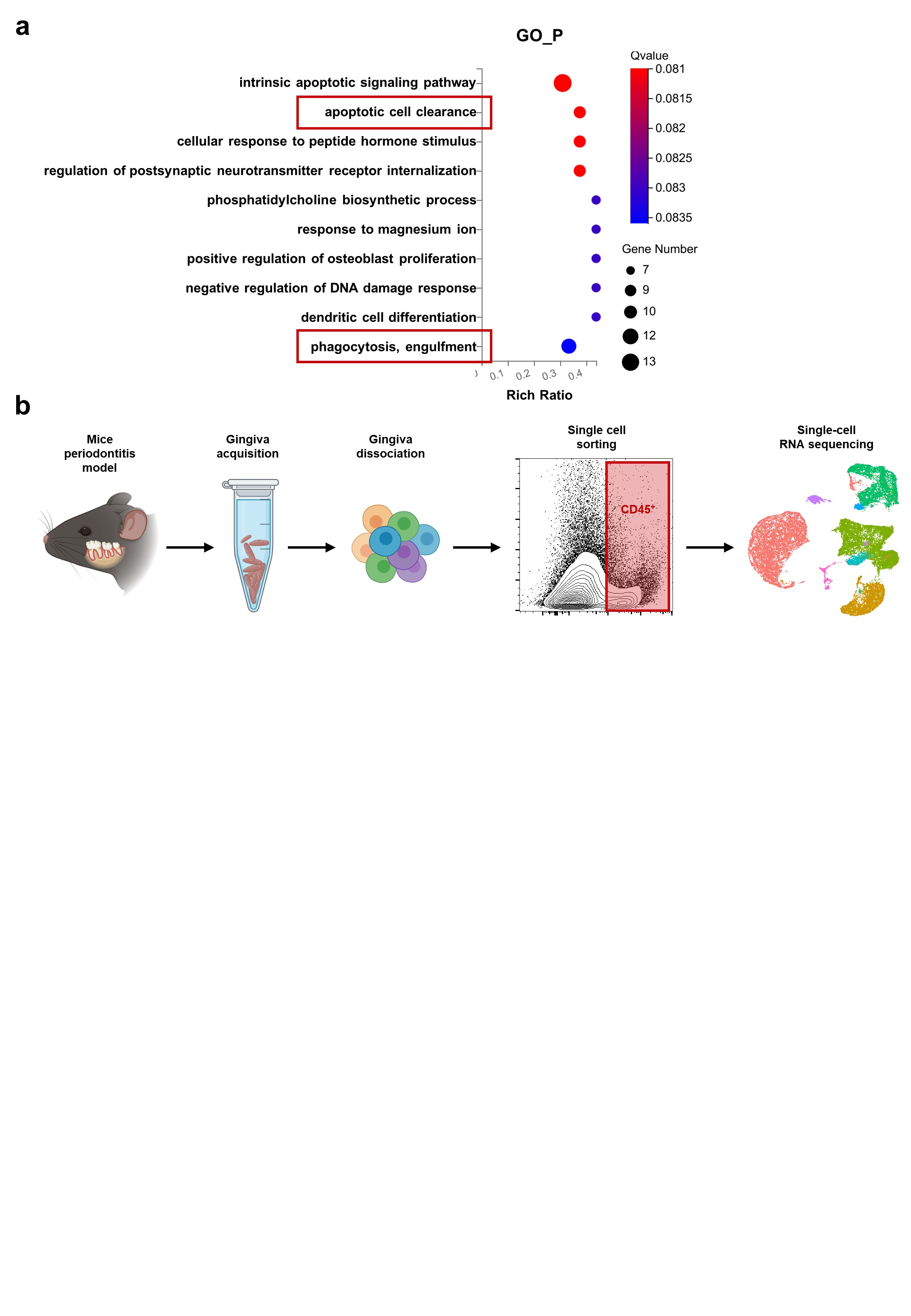


**Supplementary Figure 1**


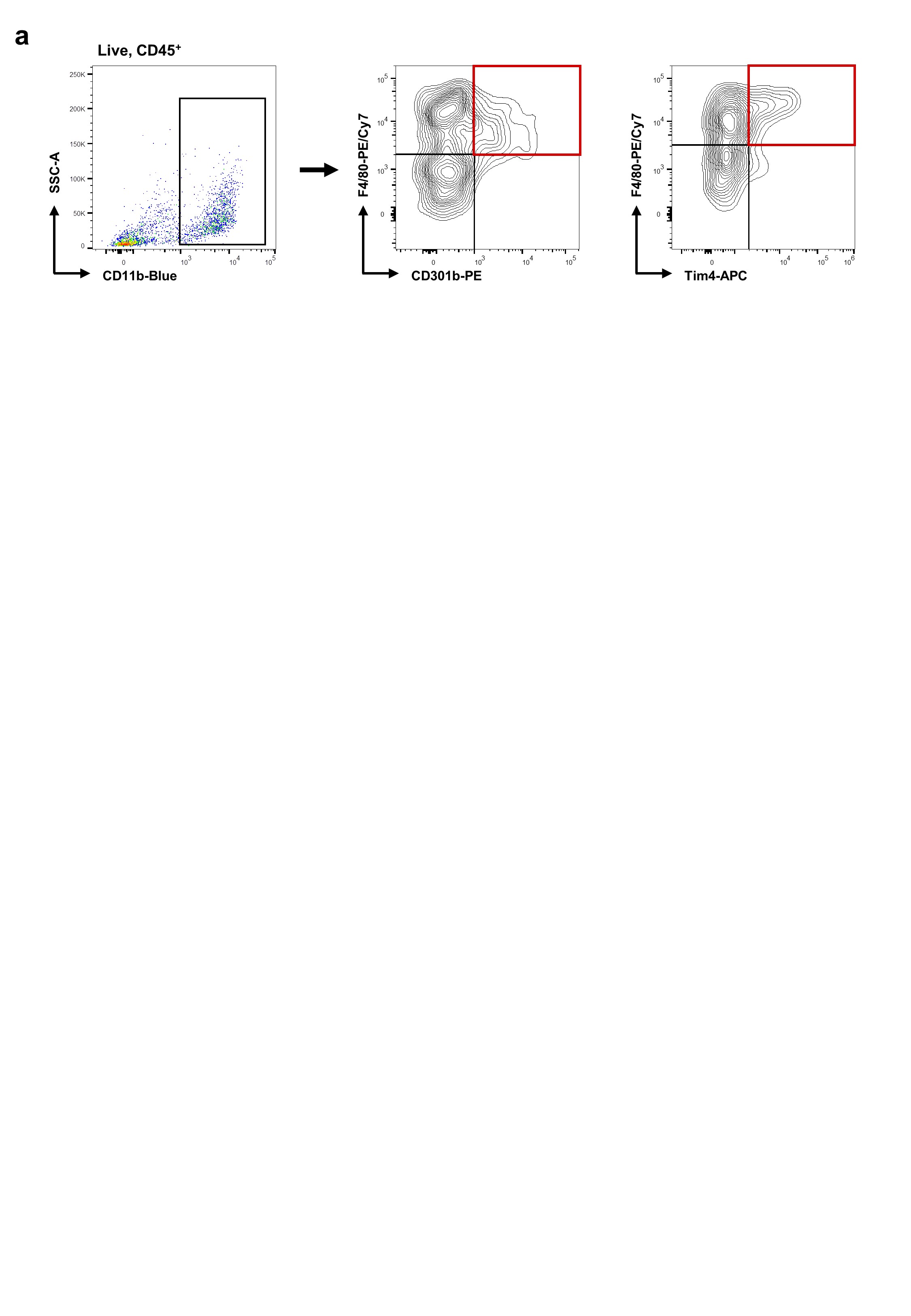


**Supplementary Figure 2**


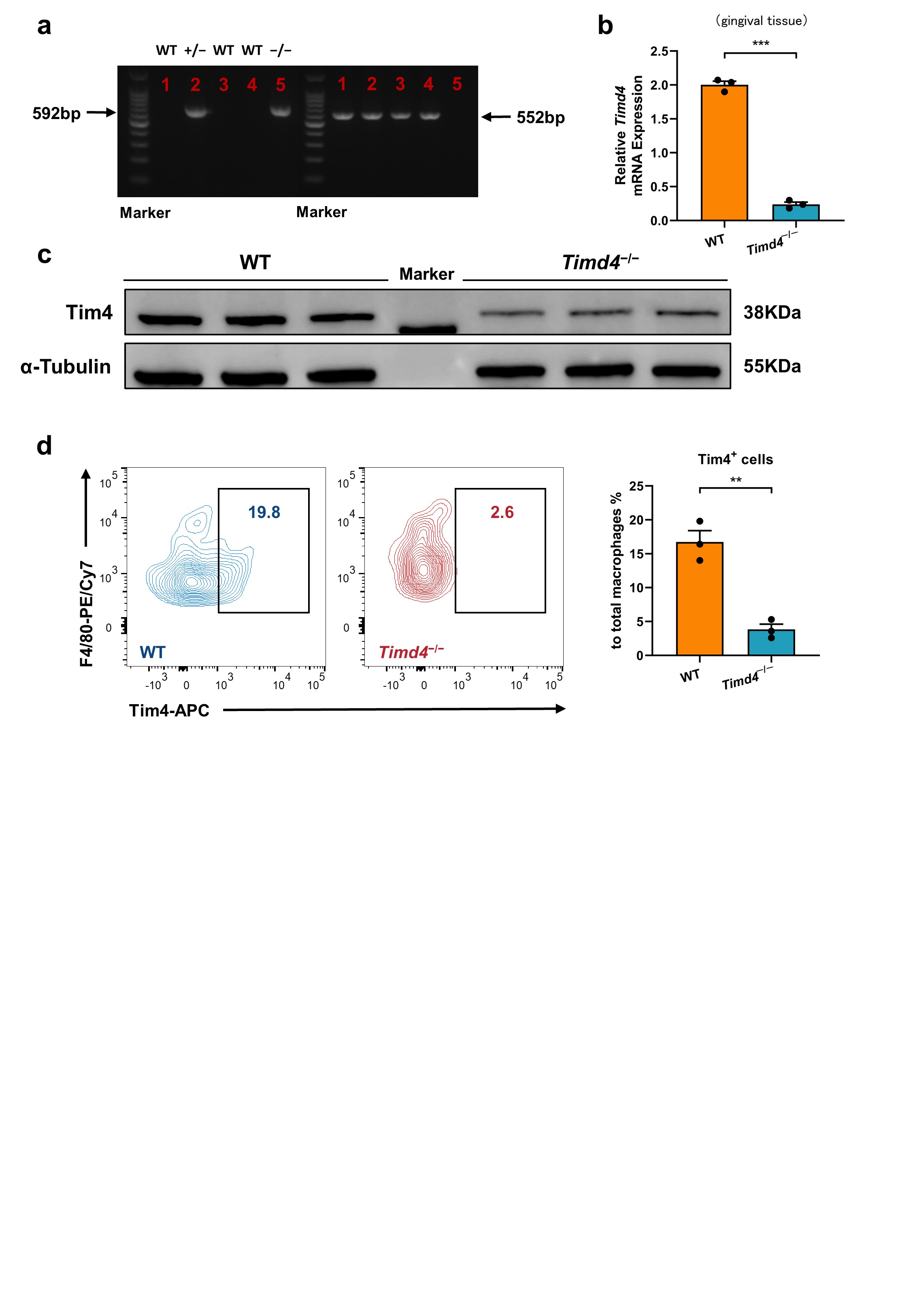


**Supplementary Figure 3**


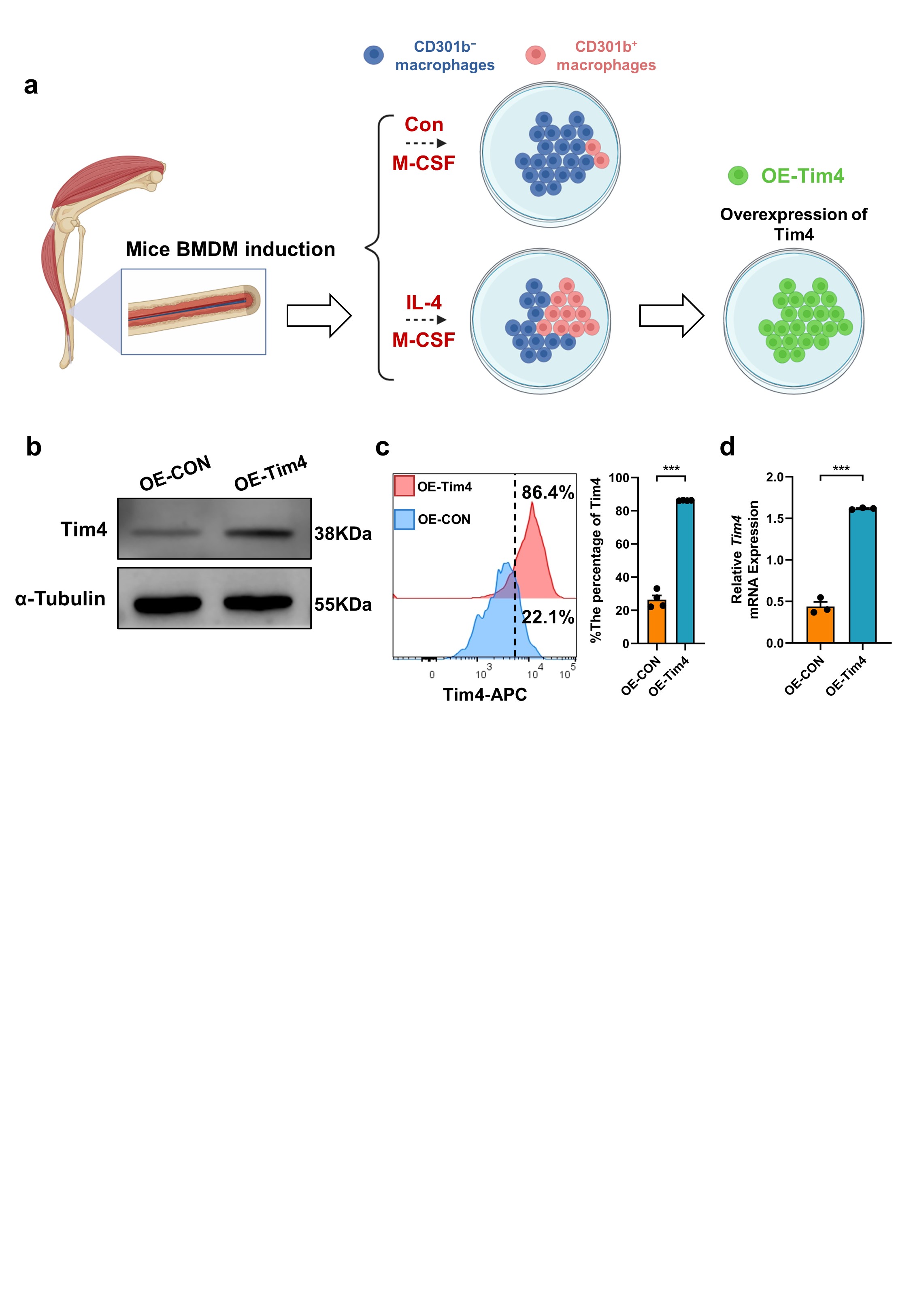


**Supplementary Figure 4**


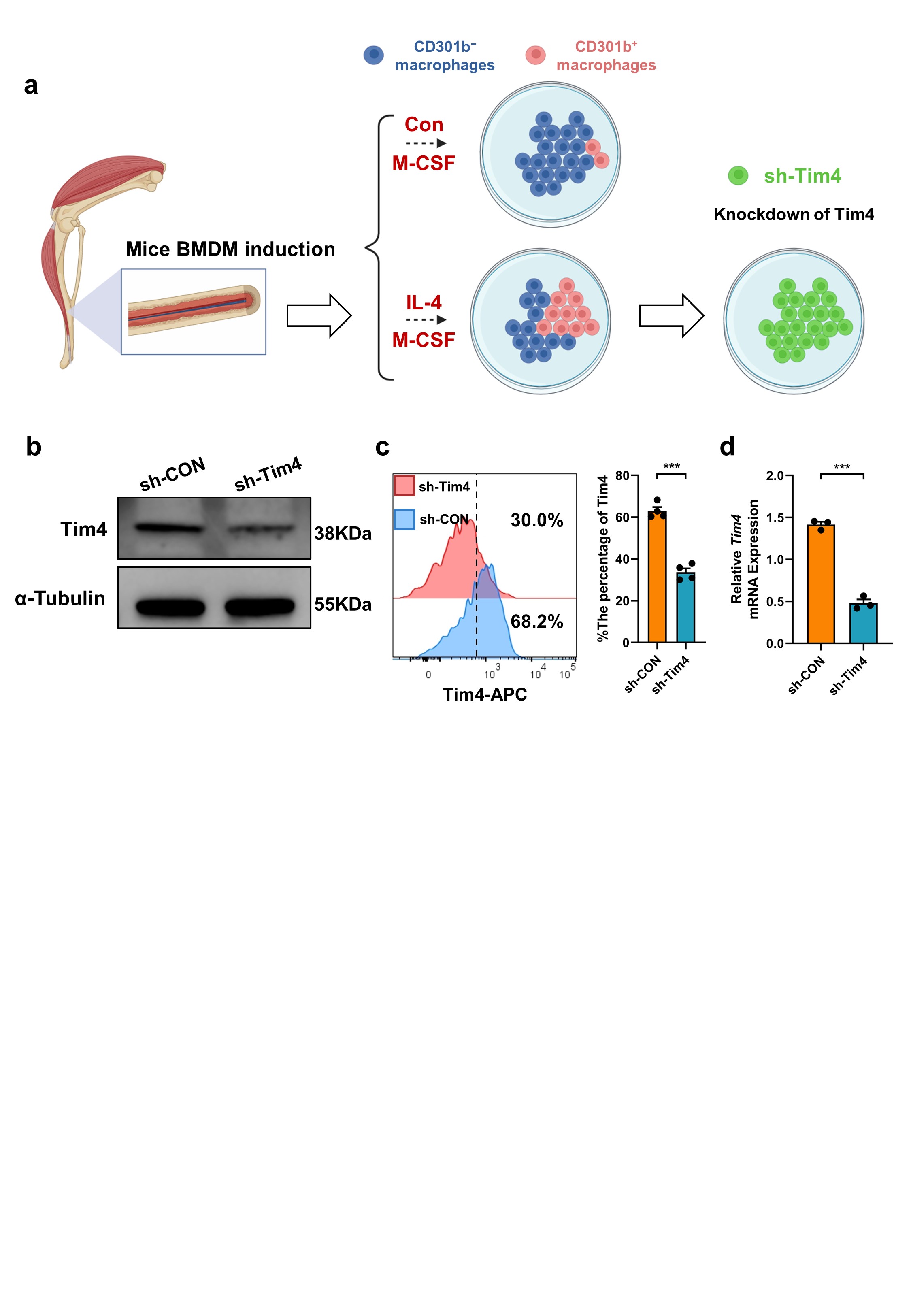


**Supplementary Figure 5**
